# Supplementary material for: Unveiling Diversity for Quality Traits in the Indian Landraces of Horsegram [Macrotyloma uniflorum (Lam.) Verdc.]
Source: Plants (Basel). 2023 Nov 8;12(22):3803. doi: 10.3390/plants12223803 (PMC10675608; doi:10.3390/plants12223803)
Supplement: Supplementary file 1 [file plants-12-03803-s001.zip › plants-2613641-supplementary.pdf]

Unveiling diversity for quality traits in Horsegram {*Macrotyloma uniflorum* (Lam.) Verdc.} landraces: A forgotten nutraceutical pulse crop of ancient India

Manju Kumari[1,2], Siddhant Ranjan Padhi[1,2], Sushil Kumar Chourey[2], Vishal Kondal[2], Swapnil S. Thakare[3], Ankita Negi[3], Veena Gupta[2], Mamta Arya[2], Jeshima KhanYasin[2], Rakesh Singh[2], Chellapilla Bhardwaj[4], Atul Kumar[4], Kailash Chandra Bhatt[2*], Rakesh Bhardwaj[2*], J C Rana[5], Tanay Joshi[6] and Amritbir Riar[6]

Supplementary Table S1. Observed values for the traits investigated in the study

S.No.	IC Number	State	Protein (g/100g)	Starch (g/100g)	Sugars (g/100g)	Phytic acid (mg/g)	Phenols (mg GAE/g)
**1**	IC432011	Maharashtra	24.15	29.81	5.94	17.00	10.40
**2**	IC432059	Maharashtra	23.29	30.25	7.94	21.15	9.06
**3**	IC278831	Himachal Pradesh	22.57	30.03	8.30	8.56	8.42
**4**	IC278832	Himachal Pradesh	23.61	29.58	9.24	10.77	9.09
**5**	IC383470	Uttarakhand	22.20	30.33	8.50	10.66	8.32
**6**	IC406524	Uttarakhand	24.73	30.44	7.76	7.05	8.69
**7**	IC392511	Uttarakhand	23.51	29.64	5.63	5.52	7.86
**8**	IC469855	Uttarakhand	23.07	29.92	5.93	7.55	9.47
**9**	IC469794	Uttarakhand	22.87	28.76	5.01	10.62	9.19
**10**	IC263263	Uttarakhand	23.60	29.29	9.27	6.87	8.45
**11**	IC263264	Uttarakhand	22.72	30.39	7.98	12.79	8.92
**12**	IC263277	Uttarakhand	22.24	29.82	7.99	13.22	8.16
**13**	IC263294	Uttarakhand	24.75	26.62	8.06	11.67	9.39
**14**	IC356050	Uttarakhand	24.13	30.97	9.34	10.35	8.80
**15**	IC382656	Uttarakhand	23.68	30.78	6.75	10.58	8.27
**16**	IC561027	Jharkhand	24.23	28.40	5.51	4.64	7.00
**17**	IC561029	Jharkhand	24.74	27.21	5.76	5.12	8.82
**18**	IC382218	Himachal Pradesh	23.24	30.34	4.95	10.79	8.04
**19**	IC361651	Himachal Pradesh	23.24	30.05	5.07	10.86	8.45
**20**	IC310660	Rajasthan	23.85	29.16	7.78	10.85	6.89
**21**	IC278825	Himachal Pradesh	23.76	27.67	6.19	7.41	11.26
**22**	IC278827	Himachal Pradesh	23.46	28.99	8.13	10.84	8.03
**23**	IC278829	Himachal Pradesh	23.92	29.99	8.90	13.92	7.04
**24**	IC278830	Himachal Pradesh	24.42	28.30	8.72	8.82	8.11
**25**	IC281653	Uttarakhand	22.80	28.85	5.69	1.88	9.24
**26**	IC281651	Uttarakhand	23.10	31.14	6.00	4.79	7.61
**27**	IC281624	Uttarakhand	23.21	30.86	8.05	5.48	8.56
**28**	IC281835	Uttarakhand	22.46	30.83	5.94	9.63	7.26
**29**	IC281820	Uttarakhand	23.24	29.94	8.02	3.41	8.38
**30**	IC469752	Uttarakhand	21.79	31.52	5.16	8.20	8.83
**31**	IC469766	Uttarakhand	21.92	32.38	8.24	11.57	8.34
**32**	IC469827	Uttarakhand	22.07	30.49	5.35	10.18	8.11
**33**	IC396009	Chattisgarh	25.06	27.70	5.85	11.08	6.64
**34**	IC396012	Chattisgarh	23.53	28.31	6.50	5.95	6.80
**35**	IC561021	Jharkhand	24.19	29.69	5.47	6.55	7.74
**36**	IC263319	Uttarakhand	23.35	30.68	5.42	11.44	7.16

S.No.	IC Number	State	Protein (g/100g)	Starch (g/100g)	Sugars (g/100g)	Phytic acid (mg/g)	Phenols (mg GAE/g)
**37**	IC263325	Uttarakhand	23.63	29.00	5.90	12.42	8.67
**38**	IC263332	Uttarakhand	23.90	31.56	5.78	10.80	9.33
**39**	IC400120	Odisha	23.72	30.65	7.90	18.68	9.38
**40**	IC385840	Jharkhand	24.15	29.17	8.74	14.95	11.02
**41**	IC385919	Jharkhand	23.44	29.06	8.19	17.70	7.98
**42**	IC385923	Jharkhand	24.16	28.07	8.06	16.22	9.59
**43**	IC391416	Uttarakhand	24.55	27.91	7.83	9.86	8.57
**44**	IC391572	Uttarakhand	24.23	31.35	8.19	11.50	8.57
**45**	IC426475	Andhra Pradesh	24.88	29.53	8.19	15.79	7.82
**46**	IC397423	Madhya Pradesh	23.98	30.30	7.92	11.01	7.40
**47**	IC397441	Madhya Pradesh	23.40	29.14	8.09	13.69	7.72
**48**	IC397443	Madhya Pradesh	24.36	30.39	5.80	18.05	6.81
**49**	IC397461	Madhya Pradesh	22.62	29.28	8.31	9.20	8.94
**50**	IC397465	Madhya Pradesh	22.14	30.90	8.64	12.36	8.48
**51**	IC397479	Madhya Pradesh	23.83	29.32	9.16	13.74	7.73
**52**	IC397511	Madhya Pradesh	24.20	29.79	10.15	9.56	5.77
**53**	IC397588	Madhya Pradesh	24.04	31.10	9.11	13.26	7.58
**54**	IC397611	Madhya Pradesh	23.51	29.91	8.58	11.13	10.22
**55**	IC423538	Himachal Pradesh	23.26	29.83	7.88	6.02	8.05
**56**	IC423572	Himachal Pradesh	22.42	31.67	8.37	16.21	6.97
**57**	IC418359	Uttarakhand	23.34	32.15	8.24	12.48	8.65
**58**	IC259884	Odisha	22.75	29.84	8.57	9.12	7.41
**59**	IC273740	Chattisgarh	22.88	29.05	8.39	5.94	9.26
**60**	IC273742	Chattisgarh	24.07	29.88	8.07	8.54	8.01
**61**	IC273743	Chattisgarh	23.52	28.18	10.69	7.16	7.75
**62**	IC273750	Chattisgarh	24.13	28.37	8.48	5.74	8.36
**63**	IC273748	Chattisgarh	21.88	32.91	8.44	16.06	7.53
**64**	IC265920	Himachal Pradesh	22.40	30.39	12.10	10.34	6.22
**65**	IC317402	Odisha	23.82	29.73	7.78	8.36	5.93
**66**	IC276108	Arunachal Pradesh	23.58	30.32	6.40	11.74	8.25
**67**	IC262082	Uttarakhand	23.74	30.14	7.57	8.04	6.50
**68**	IC262106	Uttarakhand	21.93	30.17	9.64	1.66	8.85
**69**	IC262880	Himachal Pradesh	22.53	30.91	8.35	4.56	6.77
**70**	CRHG 19	Andhra Pradesh	22.24	31.34	8.15	5.16	6.54
**71**	CRIDA 18 R	Andhra Pradesh	23.73	29.51	8.16	7.23	8.64
**72**	VL 19	Uttarakhand	23.55	28.78	7.96	7.07	8.60
**73**	BSP 1521	Chattisgarh	23.86	28.35	9.53	1.07	9.04
**74**	VL 15	Uttarakhand	23.04	30.80	8.26	7.36	8.82
**75**	IC145357	Unknown	24.20	29.99	3.16	11.96	4.39
**76**	IC139562	Telangana	22.33	32.10	3.45	8.49	6.28
**77**	IC139429	Delhi	21.77	32.00	3.99	7.47	6.97
**78**	IC023489	Chattisgarh	24.88	28.20	3.30	15.15	4.56
**79**	IC330562	Tamil Nadu	23.18	31.84	2.88	5.00	4.83
**80**	IC336492	Bihar	22.92	31.50	2.47	4.75	4.29
**81**	IC007400	Unknown	24.32	27.09	3.84	8.43	5.76
**82**	IC625403	West Bengal	25.22	28.35	3.02	5.47	7.89
**83**	IC023437	Madhya Pradesh	23.83	31.01	2.62	9.34	4.61
**84**	IC071741	Tamil Nadu	23.78	29.38	3.92	5.36	6.44
**85**	IC426522	Andhra Pradesh	24.41	28.79	3.71	8.28	7.86
**86**	IC426521	Andhra Pradesh	23.99	30.03	3.37	5.89	5.50
**87**	IC426538	Andhra Pradesh	24.66	29.08	3.47	7.65	6.61
**88**	IC426571	Andhra Pradesh	25.02	28.11	3.58	4.87	6.94

S.No.	IC Number	State	Protein (g/100g)	Starch (g/100g)	Sugars (g/100g)	Phytic acid (mg/g)	Phenols (mg GAE/g)
**89**	IC089016	Tamil Nadu	24.29	30.10	4.31	11.84	8.46
**90**	IC019431	Odisha	24.18	30.18	3.64	2.83	4.98
**91**	IC139560	Telangana	23.51	31.89	3.30	4.47	7.02
**92**	IC094617	Madhya Pradesh	22.78	30.97	2.69	5.73	5.38
**93**	IC105564	Jharkhand	22.32	31.95	4.64	10.57	3.85
**94**	IC033124	Maharashtra	23.83	30.26	2.69	9.48	6.05
**95**	IC343814	Andhra Pradesh	22.56	32.60	2.83	8.94	6.55
**96**	IC341255	Maharashtra	22.82	31.58	2.60	9.28	4.84
**97**	IC426452	Telangana	22.84	31.61	5.75	14.37	7.01
**98**	IC426464	Telangana	22.11	31.82	3.13	5.87	5.39
**99**	IC426463	Telangana	21.77	33.00	2.68	12.33	5.48
**100**	IC022772	Chattisgarh	24.95	27.00	0.86	16.71	3.96
**101**	IC022770	Chattisgarh	24.65	28.78	1.39	14.27	5.61
**102**	IC541565	Unknown	24.20	30.32	3.80	12.20	6.10
**103**	EC501564	Ethopia	24.95	29.56	3.57	11.91	8.40
**104**	IC022779A	Madhya Pradesh	23.09	31.34	3.42	7.30	4.79
**105**	IC022781	Madhya Pradesh	23.38	31.39	1.60	10.14	3.76
**106**	IC426580	Andhra Pradesh	24.18	30.52	3.20	7.03	5.14
**107**	IC426579	Andhra Pradesh	25.11	28.77	2.79	4.64	5.77
**108**	IC045726	West Bengal	23.92	30.14	3.23	11.70	5.72
**109**	IC045717A	Tamil Nadu	24.57	28.94	3.58	6.38	7.01
**110**	IC047114	Andhra Pradesh	24.79	28.27	3.92	13.25	5.85
**111**	IC049553	Maharashtra	23.49	30.83	3.80	17.05	4.80
**112**	IC044023	Kerala	25.04	27.66	4.79	11.13	6.36
**113**	IC105820	Jharkhand	23.75	31.01	3.02	18.84	5.21
**114**	IC120835	Unknown	24.27	30.40	3.04	11.64	4.57
**115**	IC071784	Tamil Nadu	22.40	29.74	3.62	11.32	6.10
**116**	IC071773	Tamil Nadu	24.39	29.81	3.20	21.21	5.60
**117**	IC139561	Tamil Nadu	24.57	30.68	2.67	5.27	5.31
**118**	IC120838	Unknown	25.20	27.74	3.23	17.12	5.15
**119**	IC123022	Unknown	23.54	28.81	3.71	11.11	5.46
**120**	IC256885	Odisha	26.15	28.49	2.59	12.80	5.17
**121**	IC328712	Andhra Pradesh	24.45	28.27	2.99	4.29	6.14
**122**	IC023482	Madhya Pradesh	26.70	28.96	1.31	14.16	3.38
**123**	IC071733	Tamil Nadu	24.39	30.60	3.20	21.21	5.60
**124**	IC044021	Kerala	26.05	28.11	3.29	19.21	4.74
**125**	IC385836	Jharkhand	23.57	30.94	2.36	16.60	5.21
**126**	IC022756	Chattisgarh	24.10	30.24	3.36	17.53	4.95
**127**	IC022840	Unknown	23.60	30.49	3.22	16.37	5.01
**128**	IC123029	Unknown	23.76	29.80	4.28	11.46	5.40
**129**	IC139512	Kerala	25.94	30.22	3.60	14.40	3.93
**130**	IC071825	Tamil Nadu	23.78	28.88	3.16	14.24	4.81
**131**	IC201115	Chattisgarh	23.75	29.31	2.49	11.79	4.70
**132**	IC022795	Chattisgarh	25.76	30.00	2.46	5.21	3.81
**133**	IC023439A	Chattisgarh	25.29	27.03	3.30	5.48	4.86
**134**	IC201108	Chattisgarh	25.76	28.75	3.01	9.38	6.96
**135**	IC071757	Tamil Nadu	24.78	28.11	3.96	8.38	5.45
**136**	IC094599A	Jharkhand	23.70	29.53	4.53	7.96	6.63
**137**	IC0369691	Andhra Pradesh	24.48	26.20	3.14	10.00	5.35
**138**	IC015735	Jharkhand	24.72	27.61	5.95	5.43	7.79
**139**	IC015728	Jharkhand	23.25	28.80	4.75	3.62	6.00

Supplementary Table S2. Extent of variance explained by principle components

Principle Component	Eigen Value	Variance %	Cumulative Variance%
**PC-1**	1.987	39.73	39.73
**PC-2**	1.430	28.59	68.33
**PC-3**	0.999	19.99	88.32
**PC-4**	0.294	5.88	94.20
**PC-5**	0.290	5.80	100.00

Supplementary Table S3. Percent contributions of the variables in principle components

SN.	Variable	PC-1	PC-2	PC-3	PC-4	PC-5
**1**	Sugars	34.96%	1.54%	5.32%	15.72%	42.46%
**2**	Starch	21.87%	8.51%	3.66%	62.70%	3.26%
**3**	Protein	14.15%	36.03%	15.77%	20.80%	13.26%
**4**	Phytic acid	1.00%	35.10%	63.44%	0.20%	0.26%
**5**	Phenols	28.02%	18.84%	11.81%	0.58%	40.76%
	Total	100.00%	100.00%	100.00%	100.00%	100.00%

Supplementary Table S4. Correlations between variables and Principle Components

S.N.	Variable	PC-1	PC-2	PC-3	PC-4	PC-5
1	Sugars	-0.78	0.13	0.23	0.35	0.45
2	Starch	-0.62	-0.30	-0.19	-0.69	0.12
3	Protein	0.50	0.62	0.39	-0.40	0.25
4	Phytic acid	0.13	-0.61	0.78	-0.04	-0.04
5	Phenols	-0.70	0.45	0.34	-0.07	-0.44

Supplementary Table S5. HCA clustering pattern and composition

States Name	No. of States Represented in cluster					Total
	I	II	III	IV	V	
Uttarakhand	**17**	**0**	**6**	**6**	**0**	**29**
Chhattisgarh	**4**	**3**	**4**	**1**	**4**	**16**
Madhya Pradesh	**4**	**2**	**2**	**5**	**1**	**14**
Andhra Pradesh	**2**	**3**	**6**	**1**	**1**	**13**
Himachal Pradesh	**8**	**0**	**2**	**2**	**0**	**12**
Jharkhand	**0**	**2**	**5**	**3**	**2**	**12**
Tamil Nadu	**1**	**3**	**3**	**0**	**3**	**10**
Unknown	**0**	**6**	**0**	**0**	**2**	**8**

Maharashtra	**0**	**2**	**0**	**2**	**1**	**5**
Odisha	**2**	**0**	**1**	**1**	**1**	**5**
Telangana	**1**	**1**	**2**	**1**	**0**	**5**
Kerala	**0**	**1**	**0**	**0**	**2**	**3**
West Bengal	**0**	**1**	**1**	**0**	**0**	**2**
Rajasthan	**1**	**0**	**0**	**0**	**0**	**1**
Arunachal Pradesh	**1**	**0**	**0**	**0**	**0**	**1**
Delhi	**1**	**0**	**0**	**0**	**0**	**1**
Bihar	**0**	**0**	**1**	**0**	**0**	**1**
Ethopia	**1**	**0**	**0**	**0**	**0**	**1**
Total	**43**	**24**	**33**	**22**	**17**	**139**

Supplementary Table S6. State-wise mean values of the traits investigated in the study

SN	State/ Country of origin	No of Accessions	Phenols mg GAE/g	Sugars g/100g	Protein g/100g	Starch g/100g	phytic acid mg/g
**1**	Andhra Pradesh	**13**	6.52	4.42	24.19	29.31	**7.92**
**2**	Arunachal Pradesh	**1**	**8.25**	**6.40**	23.58	30.32	11.74
**3**	Bihar	**1**	4.29	2.47	22.92	31.50	4.75
**4**	Chattisgarh	**16**	6.43	5.38	**24.25**	28.88	**9.82**
**5**	Delhi	**1**	6.97	3.99	21.77	32.00	7.47
**6**	Ethopia	**1**	**8.40**	**3.57**	**24.95**	29.56	11.91
**7**	Himachal Pradesh	**12**	**8.04**	**8.02**	23.24	29.81	**9.93**
**8**	Jharkhand	**12**	7.24	5.58	23.85	29.29	10.68
**9**	Kerala	**3**	5.01	3.89	**25.68**	28.66	14.91
**10**	Madhya Pradesh	**14**	6.61	6.24	23.70	30.27	11.33
**11**	Maharashtra	**5**	7.03	4.59	23.52	30.55	14.79
**12**	Odisha	**5**	6.57	6.10	24.13	29.78	10.36
**13**	Rajasthan	**1**	6.89	**7.78**	23.85	29.16	10.85
**14**	Tamil Nadu	**10**	5.96	3.45	24.01	29.81	11.02
**15**	Telangana	**5**	6.24	3.66	22.51	32.08	9.11
**16**	Unknown	**8**	5.23	3.53	24.14	29.33	12.54
**17**	Uttarakhand	**29**	**8.47**	**7.22**	23.22	30.19	**8.78**
**18**	West Bengal	**2**	6.81	3.12	**24.57**	29.25	8.59
	**Total/Overall mean**	**139**	**7.00**	**5.61**	**23.74**	**29.81**	**10.15**

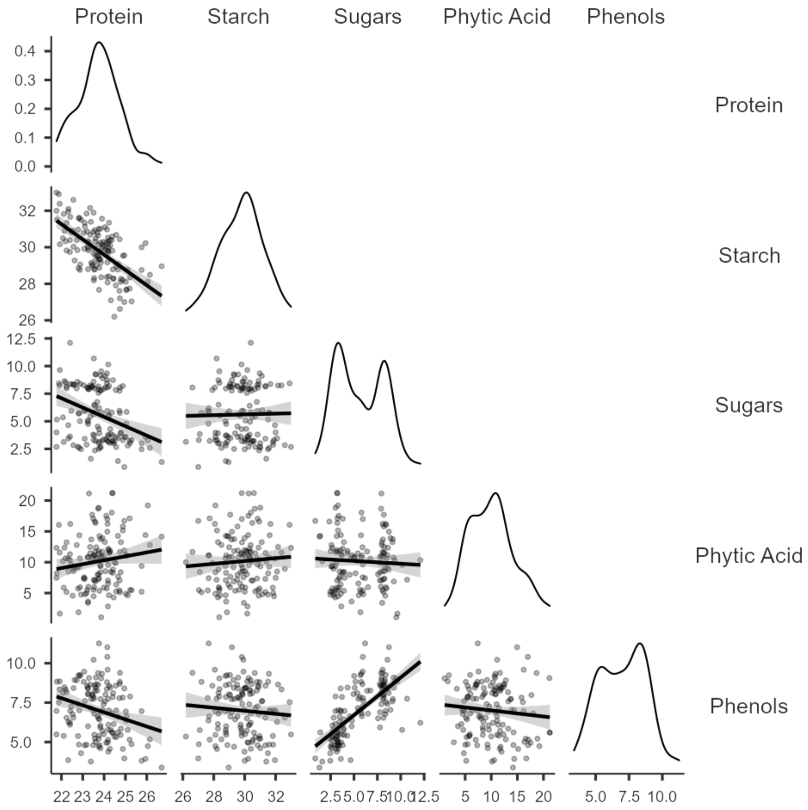

Suplimentary Fig. 1 Correlogram scatter plot matrix explaining extent of variation, data distribution and correlation of biochemicals

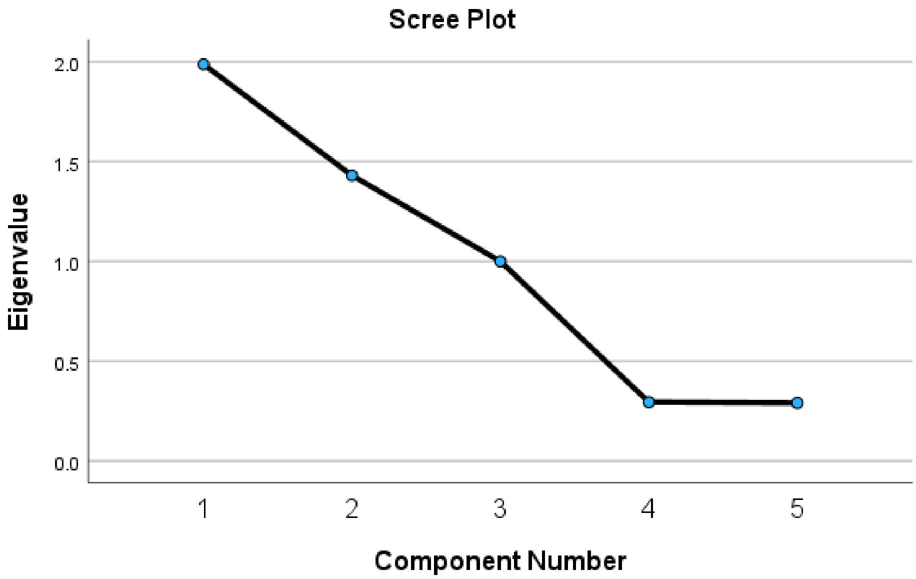

Supplementary Fig. 2 PCA Scree plot
